# Cytochrome b marker reveals an independent lineage of *Stenella coeruleoalba* in the Gulf of Taranto

**DOI:** 10.1371/journal.pone.0213826

**Published:** 2019-03-20

**Authors:** Salvatrice Ciccarese, Roberto Carlucci, Elena Ciani, Eleonora Corcella, Annalisa Cosentino, Carmelo Fanizza, Giovanna Linguiti, Rachele Antonacci

**Affiliations:** 1 Dipartimento di Biologia, Sezioni di Genetica e Biologia Animale ed Ambientale, Università di Bari “Aldo Moro”, Bari, Italy; 2 Dipartimento di Bioscienze, Biotecnologie e Biofarmaceutica, Università di Bari “Aldo Moro”, Bari, Italy; 3 Ionian Dolphin Conservation, Taranto, Italy; National Cheng Kung University, TAIWAN

## Abstract

Heterogeneity in geomorphological and hydrographical conditions throughout the Mediterranean Sea could be the driving factors behind the significant differences between putative sub-populations, although the existence of a large panmictic population of striped dolphin *Stenella coeruleoalba* (Meyen 1833) in this marine region could not be excluded. However, understanding the ecological implications of such genetic differentiation is difficult, as inferences about gene flow are usually made on evolutionary time scales and not along the ecological time frame over which most management and conservation practices are applied. In fact, as stated by the IUCN Red List, in the case of species assessed as vulnerable, the degree of genetic exchange between populations within a biogeographic region and its ecological implications represent a fascinating challenge that should be very deeply explored. This is even more significant in the Gulf of Taranto (Northern Ionian Sea, Central-eastern Mediterranean Sea), where the geomorphological and hydrographic characteristics support the hypothesis of a separated striped dolphin population genetically diverging from its original Mediterranean counterpart. To assess this hypothesis, a genetic analysis was carried out on DNA fragments of the mitochondrial *cyt b* gene to explore the evolutionary origin of *S*. *coeruleoalba* in the investigated area and its genetic diversity in comparison with available sequences from other Mediterranean and Atlantic populations. Results were discussed indicating ecological implications and suggesting conservation objectives. Moreover, a delphinid systematic was also suggested.

## Introduction

Cetaceans, as marine mammals, diverged from their terrestrial ancestors approximately 54 million years ago [1]. They are phylogenetically placed in the Cetartiodactyla clade, which includes Cetacea and Artiodactyla. The close relationship between marine and terrestrial mammals is proven by both morphological data and molecular analysis [2]. In fact, the recent genome assemblies have made it possible to compare both whole genomes [3] and large and sizable portions of them [4].

In Cetacea, particularly within the Delphinidae family, the evolutionary relationships among dolphin species are not yet well understood nor in complete agreement because of a recent and rapid species radiation [5, 6]. Above all, the genus *Stenella* can be considered an artificial assemblage within the family Delphinidae, with some member species (i.e. *Stenella coeruleoalba* Meyen, 1833) more closely related to *Tursiops*, *Delphinus*, *Sousa*, or *Lagenodelphis* than to nominal congeners [5, 6, 7].

The striped dolphin *S*. *coeruleoalba*, which is distributed in tropical and temperate pelagic waters [8], is the most frequently observed cetacean both inshore and offshore of the Mediterranean Sea [9], with sightings decreasing westward [10, 11, 12].

Despite heterogeneity in geomorphological and hydrographical conditions throughout the Mediterranean Sea [13] that were confirmed as driving Cetacean’s distribution [14, 15, 16], there is little evidence for population structure in the striped dolphin [17], and small but significant differences between the putative populations were indicated by Gaspari et al. [11]. However, although the existence of a large panmictic population of striped dolphin in the Mediterranean Sea could not be excluded [18], the degree of genetic exchange, and its ecological implications should be very deeply explored. In fact, understanding the ecological implications of such small, but significant, genetic differentiation in population structure is difficult because of gene flow inferences that usually occur on evolutionary time scales, not along the ecological time frame over which most management and conservation practices are applied [19, 20, 21]. In addition, the UE Marine Strategy Framework Directive (MSFD, Directive 2008/56/EC) requires an assessment of the status of the marine environment, in relation to the main pressures caused by human activities, to achieve an evaluation of ‘Good Environmental Status’ by 2020. This formally includes an assessment of the status of cetacean species. An analysis of the population genetic structure might help clarify possible connectivity, and identify distinct management units for conservation, to facilitate the cetacean assessment. This is significant in the Gulf of Taranto in the Northern Ionian Sea (Central Mediterranean Sea), where the striped dolphin, as well as other cetacean species, could be exposed to elevated levels of anthropogenic threats [22, 23, 24].

In this study, genetic and evolutionary analyses were carried out to test the hypothesis of a local population of striped dolphin in the Gulf of Taranto (Northern Ionian Sea, Central-eastern Mediterranean Sea). The hypothesis is favoured by the peculiar eco-physiographic features as the presence of a submarine canyon system, the mixing of surface and dense bottom waters as well as the occurrence of variability in upwelling currents [25, 26, 27, 28]. All these features make it possible to feed this top predator of mesopelagic fish, cephalopods and planktonic crustaceans [29, 30, 31]

Specifically, DNA fragments of the mitochondrial *cyt b* gene were sequenced to study the evolutionary origin of *S*. *coeruleoalba* in the study area, as well as to compare genetic diversity and the relationship with other available sequences from Mediterranean and Atlantic populations.

## Materials and methods

### Study area

**Fig 1:**
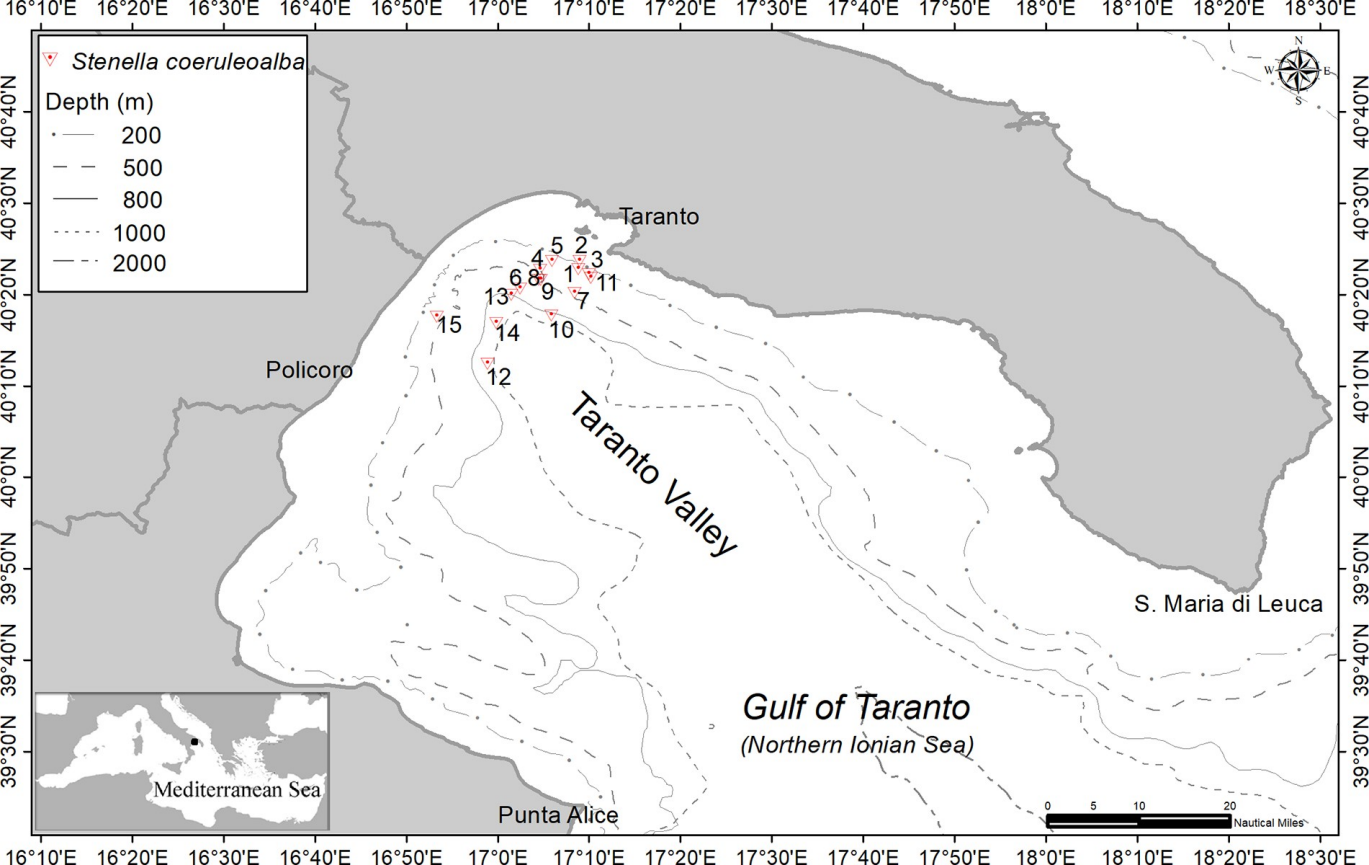
Map of the Gulf of Taranto (Northern Ionian Sea, Central-eastern Mediterranean Sea) indicating the survey area. Red triangles indicate the fifteen sampling groups sites (see Table 1).

The Gulf of Taranto, in the Northern Ionian Sea (Central-eastern Mediterranean Sea), covers an area of approximately 14,000 square kilometres from Santa Maria di Leuca to Punta Alice showing a very complex topography (Fig 1). A narrow continental shelf, with a steep slope and several channels, characterize the western sector, while the eastern sector shows terraces descending toward the “Taranto Valley”. The Taranto Valley is a NW-SE submarine canyon with no clear bathymetric connection to a major river system [25, 26, 27, 28]. This singular morphology involves a complex distribution of water masses, where the mixture of surface and dense bottom waters in upwelling current varies seasonally [22, 23, 24, 32, 33].

### Samples collection and ethics statement

Skin tissue from striped dolphins were sampled under authorizations provided by the CITES Regional Services (Register n. 6263/2009/PAB_CFS) during 15 standardized vessel-based surveys carried out from April to August 2017 by using a 12-m catamaran sailing from the harbour of Taranto or Policoro (Table 1 and Fig 1). The investigated area was in the northernmost part of the Taranto Valley extending on a bathymetric range between 175 to 900 m in depth. The number of individuals sampled during each day varied between 1 and 6 for a total of 57 striped dolphins. Within each survey day the sampled dolphins belonged to the same group. The group size in the striped dolphin sampled population fluctuated from 10 to 250 individuals.

**Table 1:** Sampling details, haplotypes and sex determination of *S*. *coeruleoalba* in the Gulf of Taranto.

Sampling group and date ^a^	Lat. in decimal degree (N)	Lon. in decimal degree (E)	Depth (m)	Estimated pod size	Sampling code	DNA	Haplotype^b^	Sex
**1°** (4–27)	40.378767	17.147067	282	40	1	**-**	**-**	**-**
					2	**-**	**-**	**-**
					**3**	**+**	**Hap10**	**nd**
					4	**-**	**-**	**-**
					**5**	**++**	**Hap10**	**M**
**2°** (4–30)	40.393967	17.149900	175	200	**6**	**++**	**Hap10**	**F**
					7	**-**	**-**	**-**
					8	**-**	**-**	**-**
**3°** (5–1)	40.369517	17.166683	240	200	**9**	**+**	**Hap10**	**nd**
					**10**	**++**	**Hap10**	**M**
					**11**	**++**	**Hap10**	**F**
**4°** (5–2)	40.378017	17.077633	500	50	**12**	**++**	**Hap10**	**F**
					13	**-**	**-**	**-**
					14	**-**	**-**	**-**
					15	**-**	**-**	**-**
					**16**	**++**	**Hap10**	**M**
					17	**-**	**-**	**-**
**5°** (6–3)	40.393783	17.099067	270	30	18	**-**	**-**	**-**
					**19**	**++**	**Hap10**	**F**
					20	**-**	**-**	**-**
					21	**-**	**-**	**-**
**6°** (6–6)	40.343133	17.041667	650	100	**22**	**++**	**Hap3**	**M**
					23	**-**	**-**	**-**
					**24**	**++**	**Hap4**	**M**
					25	**-**	**-**	**-**
					**26**	**++**	**Hap5**	**M**
**7°** (6–12)	40.33495	17.140200	450	200	**27**	**-**	**-**	**-**
					**28**	**-**	**-**	**-**
					**29**	**-**	**-**	**-**
					**30**	**-**	**-**	**-**
					**31**	**++**	**Hap1**	**F**
					32	**-**	**-**	**-**
**8°** (6–14)	40.36005	17.075850	590	30	**33**	**+**	**Hap2**	**nd**
					34	**-**	**-**	**-**
**9°** (6–25)	40.359017	17.078433	510	250	**35**	**+**	**Hap10**	**nd**
					**36**	**+**	**Hap10**	**nd**
**10°** (6–27)	40.294917	17.098917	900	80	37	**-**	**-**	**-**
					38	**-**	**-**	**-**
					**39**	**++**	**Hap4**	**M**
**11°** (7–1)	40.363017	17.170833	270	25	**40**	**++**	**Hap5**	**M**
					41	**-**	**-**	**-**
					42	**-**	**-**	**-**
					43	**-**	**-**	**-**
**12°** (7–5)	40.2066	16.982250	900	30	44	**-**	**-**	**-**
					**45**	**++**	**Hap11**	**M**
**13°** (7–10)	40.331567	17.024867	700	10	**46**	**++**	**Hap5**	**M**
					**47**	**++**	**Hap6**	**M**
					**48**	**++**	**Hap5**	**M**
					**49**	**++**	**Hap7**	**M**
					50	**-**	**-**	**-**
					51	**-**	**-**	**-**
**14°** (8–7)	40.280983	16.997633	750	50	**52**	**++**	**Hap8**	**M**
**15°** (8–17)	40.292533	16.889450	500	40	53	**-**	**-**	**-**
					54	**-**	**-**	**-**
					55	**-**	**-**	**-**
					**56**	**++**	**Hap9**	**M**
					57	**-**	**-**	**-**

DNA:—<200 ng; + <300 ng; ++ >300 ng; Sex: nd (not detectable). The samples used in this study are in bold. The amount of extracted DNA, the name of each haplotype and the sex determination (where possible) are also indicated.

(a) starting date: 4-27-2017; ending date: 8-17-2017.

(b) The Hap numbers were assigned on the basis of the processing order of the DNA samples.

Considering that striped dolphins are used to approaching boats during sightings, the skin swabbing, a less invasive method for the tissue sampling, was employed rather than puncturing [34, 35, 36]. A moderately abrasive 4 X 4 cm synthetic fibre scrub pad was attached with plastic fasteners to the tip of 130 cm long wooden sticks, was used to collect the skin samples [37]. The skin tissue samples were removed from an individual dolphin by friction with a scrub pad on its back, then transferred to a flask containing a 70% alcohol solution. The striped dolphins reacted to the skin swab, swimming faster, jumping, or diving after contact. However, sometimes they returned close to the boat, confirming that the method did not cause any irreversible stress. Sampled individuals were clearly distinguishable based on the light skin scratches caused by swabbing, thus preventing unwanted resampling of the same dolphin.

### DNA extraction, PCR amplification, and sequencing

The skin adhered in the scrub pad was removed and DNA was extracted using Chelex-100 (Sigma) resin suspension in TE (10 mM Tris HCl, 1 mM EDTA, pH 8.0). Chelex solution (500 μL at 15%) was added to tubes containing the skin specimen trapped in the fibre scrub pad. The tubes were vortexed and incubated at 100° C for 15 min, and then placed on ice for 2 min. The fibre scrub pad was then removed from the tube with a sterile gripper. The skin samples were centrifuged at 1300 rpm for 5 min, and the supernatant was transferred to new tubes. The DNA was purified by a standard phenol/chloroform method, quantified, and the quality checked with a Nanodrop 1,000 spectrophotometer from Thermo Scientific.

To ensure the uniqueness of the samples from individuals that share the same haplotype (see for example the sampling group 3 in Table 1), a protocol for sex determination and photographic material (S1 Fig as an example) were applied. For sex determination, genomic fragments of the *S*. *coeruleoalba ZFX/ZFY* and *SRY* genes were amplified. We designed one set of three oligonucleotide primers for multiplex PCR amplification of the *ZFY* and *ZFX* sequence. The set consisted of a forward-orientated oligonucleotide primer designed to anneal to the *ZFY*, as well as the *ZFX* sequence (ZFYX0582F, 5’-ATAGGTCTGCAGACTCTTCTA-3’), and two reverse-orientated oligonucleotide primers placed within a polymorphic position between the *ZFX* (ZFX0923R 5’-AGAATATGGCGACTTAGAACG-3’) and *ZFY* sequences (ZFY00767R 5’-TTTGTGTGAACTGAAATTACA-3’). The multiplex PCR reaction consisted of 300 ng of sample DNA, 1 U of Taq polymerase—Platinum (Life Technology), 10 μM of each primer, 10 mM dNTPs, 50 mM MgCl_2_, and 10X PCR buffer to a final volume of 25 μl. The PCR conditions were: 2 min at 94°C for pre-heating, 35 cycles of 94°C for 60 seconds, 56°C for 30 seconds, 72°C for 30 seconds, and 72 C° for 5 min post-extension in an automated thermal cycler. All reaction products were electrophoresed on 2% agarose gels containing 0.5 μg/ml ethidium bromide and documented with a gel electrophoresis visualization system. The expected product size was a 382 bp single band, as determined by electrophoresis, for females and two 382 bp and 226 bp bands for males. Another primer set (SRYF 5’-GAATATTCCCGCTCTCCGGAG-3’, SRYR 5’- ACCTGTTGTCCAGTTGCACT-3’) was used to amplify a 418 bp fragment of the *SRY* gene. The PCR conditions were those described above. In some samples, sex determination was not carried out owing to the scarcity of DNA sample (“nd” in Table 1).

For the mitochondrial DNA (mtDNA), a fragment of the *cyt b* gene was amplified in a volume of 25 μl, using Taq polymerase—Platinum (Life Technology) by two consecutive PCR reactions. For the first reaction, the primer sets (F1cytB 5’-TAACAGTCATGGCCACTGCATT-3’ and R2cytB 5’-TGGTTTGATGTGTGCAGGGGTG-3’) were used under the following conditions: 500 ng of each DNA sample, 10 mM dNTP, 50 mM MgCl2, 1 U Taq, 10 μM of each primer, and 10X PCR buffer. The PCR program has an initial cycle of 94°C for 4 min, 35 cycles of 94°C for 15 s, 60°C for 30 s, 72°C for 30 s, and 72°C for 5 min. The PCR conditions for the second semi-nested PCR reaction using the F1cytB and R1cytB (5’-AGGGTGGAATGGAATTATGTCT-3’) primers were set as described above. The PCR program included an initial cycle of 94°C for 4 min, 35 cycles of 94°C for 15 s, 62°C for 5 s, 72°C for 30 s, and 72°C for 5 min. The PCR products were purified and full-sequenced, in both directions, by a sequencing commercial service, using another forward primer (F2cytB 5’-CCAACCTCTTATCAGCAATC-3’) along with the same reverse primer (R1cytB) used in the semi-nested PCR. A forward primer (F2cytB) drawn downstream of the forward primer used in the semi-nested PCR was using for sequencing, which allowed us to proceed directly to the sequencing of PCR products from fragments.

### Genetic analyses

**Fig 2:**
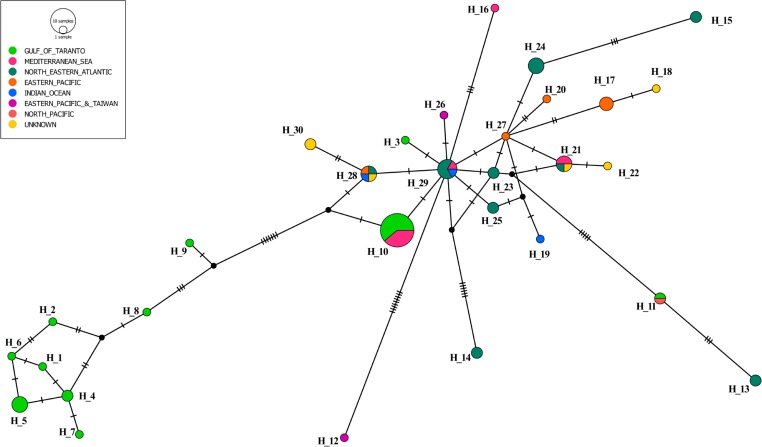
Median-joining network of cytochrome b haplotypes from *S*. *coeruleoalba*. Circle size is proportional to the number of individuals exhibiting that haplotype (H_1 represents a single animal and H_10 represents 18 animals). Colours indicate the geographic origin of the samples. Mutation events separating haplotypes are indicated as "hatch marks". Black circles indicate missing, intermediate haplotypes.

The mtDNA sequences were aligned using the *Clustal Omega* software (EBI). The *cyt b* gene analysis was conducted on a partial sequence 421 bp in length. The number of haplotypes was derived with the software DnaSP version 5.10.01 [38]. The haplotype and nucleotide diversity, number, and type (single variable and parsimony information sites; synonymous and replacement changes) of single nucleotide polymorphisms were also assessed using DnaSP. The phylogenetic network, based on 421 bp *cyt b* gene sequences from 74 *Stenella coeruleoalba* (S1 Table) from the study area and other geographic sampling sites (Fig 2), was constructed with a median-joining algorithm and implemented in the PopART (Population Analysis with Reticulate Trees) software [39] using Median Joining Network (MJN) method.

Additionally, we designed a dataset of 94 nucleotide sequences (only one sequence for each haplotype) retrieved from GenBank, including *S*. *coeruleoalba* from the Gulf of Taranto (this work), *S*. *coeruleoalba* from other geographic areas (Table 2), *S*. *clymene*, *S*. *frontalis*, *S*. *longirostris*, *S*. *attenuata*, *T*. *aduncus*, *T*. *truncatus*, and *D*. *delphis*. The accession numbers of all sequences are listed in S1 and S2 Tables. We constructed a phylogenetic tree from the above dataset using the neighbour-joining (NJ) [40] and the maximum likelihood (ML) [41] methods implemented in MEGA 7 [42]. For the phylogenetic analysis, multiple alignments were carried out for the *cyt b* gene sequences with the MUSCLE program [43]. The evolutionary distances were computed using the p-distance method [44] (NJ tree) and the maximum likelihood method based on the Tamura-Nei model [41] (ML tree), and the units are in the number of base differences per site.

**Table 2:** Nucleotide and haplotype diversity in *Stenella coeruleoalba* mtDNA cytochrome b sequence.

Groups	N° of sequences	Nucleotide diversity	Polymorphic sites	Singleton variable sites	Parsimony sites	Haplotype diversity	N° of haplotypes	Synonymous changes	Replacement changes
Gulf of Taranto	25	0.01990±0.00139	23	6	17	0.793±0.076	11	23	0
Mediterranean	11	0.00406±0.00133	6	3	3	0.600±0.154	4	6	0
Gulf of Taranto plus Mediterranean	36	0.01797±0.00205	26	7	19	0.744±0.076	14	26	0
NE Atlantic	20	0.01176±0.00232	20	1	19	0.911±0.034	9	20	0
Eastern Pacific	6	0.00602±0.00151	6	4	2	0.800±0.172	4	4	2
Combined groups	74*	0.01684±0.00180	46	14	32	0.925±0.022	30	44	2

*The number includes also the sequences from Maldives (3), Taiwan (2), North Pacific (1) and unknown geographic provenience (6).

## Results

### Population analysis

Skin swabs from striped dolphins were confirmed as an applicable method for minimized invasion allowing tissue collection for genetic analysis with an acceptable level of adequacy. The swabbing of skin allowed us to collect an acceptable amount of tissue: DNA yield between 200 and 300 ng were obtained from 25 of 57 samples, useful for the PCR amplification and sequencing (Table 1).

A 421-bp piece of the mtDNA cytochrome b gene was successfully amplified and sequenced in 25 *S*. *coeruleoalba* individuals (Acc. Numbers from LT971396 to LT971420). The sequences were designated with the initials “Hap” and a progressive number and were aligned using the *Clustal Omega* software (EBI). 11 *S*. *coeruleoalba* haplotypes were obtained from the study area (Table 1). Hap 10 was the most frequent, being represented in 11 of the 25 dolphin individuals, and it was followed by Hap 5 (4/25) and Hap 4 (2/25). The remaining eight haplotypes were found in single individuals. The haplotypes had 23 polymorphic sites, including 6 single variable sites (34, 280, 286, 295, 388 397 bp) and 17 parsimony information sites (91, 94, 109, 130, 178, 181, 184, 217, 244, 250, 259, 304, 310, 329, 341, 350, 394 bp) (Table 3). Our sequences were assessed against 49 sequences from GenBank for *S*. *coeruleoalba* to give a total of 74 sequences. All sequences were aligned by *Clustal Omega* software (EBI), allowing us to identify 19 new haplotypes (named from Hap 12 to Hap 30) (S1 Table). The sequences were grouped according to their area of origin, such as *S*. *coeruleoalba* from the Gulf of Taranto (25 in this study), those coming from other parts of the Mediterranean Sea (11), North-Eastern Atlantic (20), the Eastern Pacific (6), Indian Ocean (Maldives, 3), Eastern Pacific (Taiwan, 2), North Pacific (1), and an unknown geographic provenance (6). The location of origin for the GenBank sequences were verified with reference to the published paper [7, 36] or by direct correspondence with the author. Table 2 shows the nucleotide and haplotype diversity of the most numerous *Stenella* groups such as the Gulf of Taranto (Northern Ionian Sea, Central-eastern Mediterranean Sea), North-Eastern Atlantic, Eastern Pacific, and the combined groups. Of the 421 bp analysed, 46 were informative sites. The majority were at the 3rd-codon-position-site (synonymous changes), with only two replacement changes, found both in the Eastern Pacific group.

**Table 3:** Haplotypes identified in the partial (421 bp) mitochondrial cytochrome b gene sequence, along with sample size. The position in the sequence where the substitution occurred is numbered in the header. Nucleotide positions underlined represent single variable sites.

	No. of sequences	34	91	94	109	130	178	181	184	217	244	250	259	280	286	295	304	310	329	341	350	388	394	397
**Hap-1**	1	A	C	C	C	T	T	T	A	A	C	C	C	C	C	T	C	C	T	T	T	T	C	C
**Hap-2**	1	-	-	-	A	-	-	-	-	G	-	-	-	-	-	-	-	-	-	-	-	-	T	-
**Hap-3**	1	G	T	T	-	C	C	C	G	G	T	A	T	-	-	-	T	T	C	C	C	-	T	-
**Hap-4**	2	-	T	-	-	-	-	-	-	-	-	-	-	-	-	-	-	-	-	-	-	-	-	-
**Hap-5**	4	-	T	-	A	-	-	-	-	-	-	-	-	-	-	-	-	-	-	-	-	-	-	-
**Hap-6**	1	-	-	-	A	-	-	-	-	-	-	-	-	-	-	-	-	-	-	-	-	-	-	-
**Hap-7**	1	-	T	-	-	-	C	-	-	-	-	-	-	-	-	-	-	-	-	-	-	-	-	-
**Hap-8**	1	-	T	-	-	-	-	-	-	G	-	-	-	-	-	-	-	-	-	-	C	-	T	-
**Hap-9**	1	-	T	-	-	-	C	C	-	G	T	-	-	-	-	-	-	-	-	-	C	-	T	T
**Hap-10**	11	-	T	T	-	C	C	C	G	G	T	A	T	-	-	-	T	T	C	-	C	-	T	-
**Hap-11**	1	-	T	T	-	C	C	C	G	G	T	-	T	T	T	C	-	T	C	C	C	C	T	-

Levels of genetic diversity were high for all groups of *S*. *coeruleoalba*, with the population from the Gulf of Taranto showing the highest nucleotide diversity (Table 2).

In particular, the nucleotide diversity for the 25 samples from the Gulf of Taranto (0.01990±0.00139) is higher than the value observed in the combined group containing the Gulf of Taranto and the Mediterranean Sea (0.01797±0.00205). Nevertheless, this value is also higher than that of the nucleotide diversity observed in all the combined groups (0.01684±0.00180). This result suggests remarkable heterogeneity that characterizes the sample set of the study area (Table 2).

### Phylogenetic analysis

The median joining haplotype network showed a clear separation between eight haplotypes observed, for *S*. *coeruleoalba* in the study area (green circles in the lower part of Fig 2), and all the remaining sequences. The grouping of the eight haplotypes (Hap 1, 2, 4, 5, 6, 7, 8, and 9), representing 12 samples, differed considerably from Hap 10, representing 11 samples from which it is separated by at least nine substitutions, the closest being Hap 9. It is worth noting that the most represented haplotype (the largest circle in Fig 2) in the Gulf of Taranto (Hap 10) was shared by striped dolphins that originated from the Mediterranean Sea. Similarly, Hap 3 and Hap 11 are compatible with a Mediterranean origin, as both are linked to at least one haplotype (Hap 29 and/or Hap 21) found in some subjects sampled in this basin (Amaral, personal communication).

The network analysis indicates that the four haplotypes from the Mediterranean Sea (Hap 10, 16, 21 and 29) are related to each other, as they are separated by a limited number of substitutions. It is also possible to observe a certain geographical gradient as the most common haplotype in the Mediterranean (Hap 10) emerged directly from Hap 29 with a single parsimony information site (pos. 341). The main component of Hap 29 is derived from North-Eastern Atlantic. Similarly, Hap 16 and Hap 21 resulted directly connected to Hap 29.

Finally, the network topology and the frequency pattern shown in Fig 2 indicated that Hap 29 occupied a central position. Notably, Hap 29 is located at a junction between the four haplotypes (Hap 17, 20, 27 and 28) from the Eastern Pacific. Taken together, the above observation, and the Hap 29 star-like appearance, are suggestive of an expanding population hypothesis.

**Fig 3:**
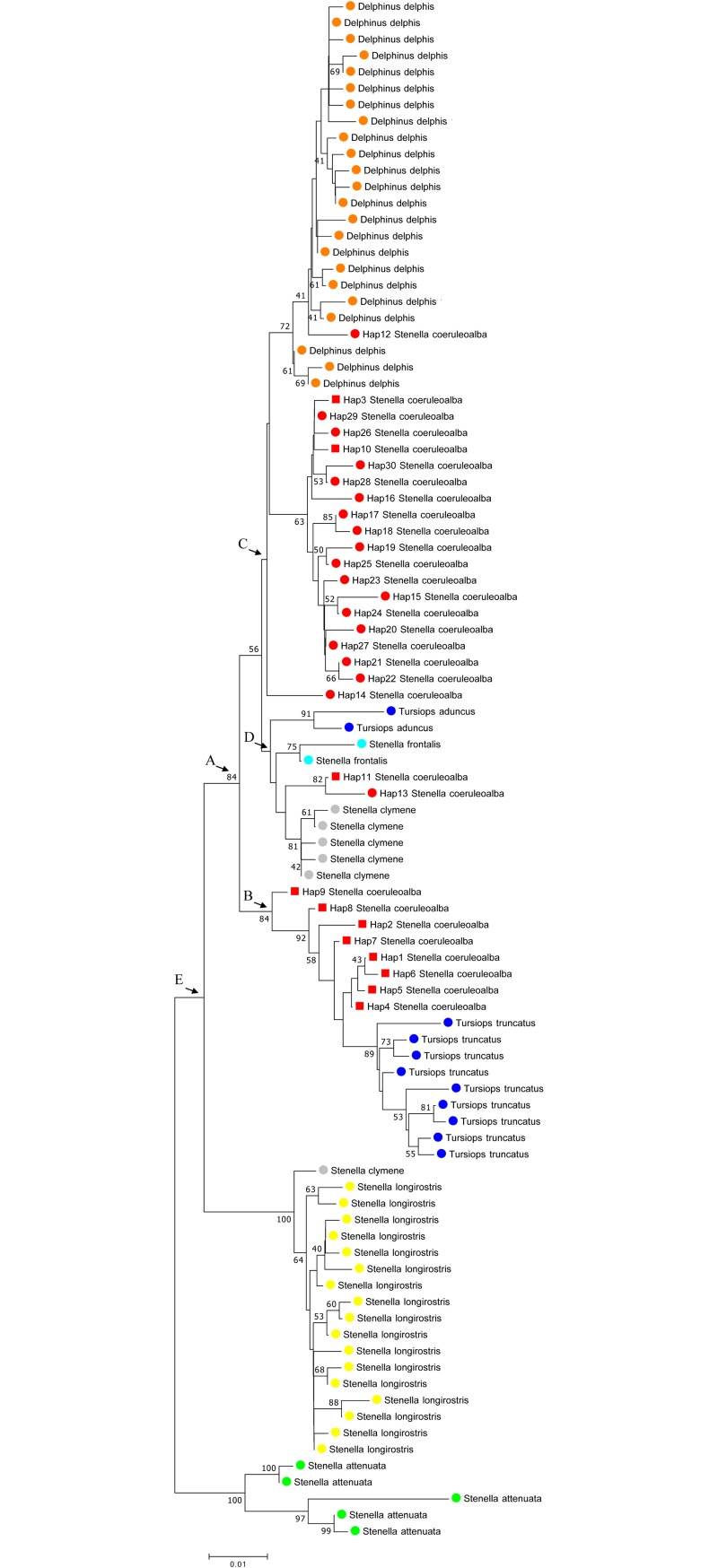
NJ tree inferred from Delphinidae *cyt b* gene sequences. Evolutionary analysis was conducted using MEGA7 [42]. The optimal tree, with a sum of branch length = 0.45049170, is shown. The tree is drawn to scale with branch lengths in the same units as those of the evolutionary distances used to infer phylogenetic trees. The evolutionary distances were computed using the p-distance method [44] and are in the units of the number of base differences per site. The analysis involved 95 nucleotide sequences. Codon positions included were 1st+2nd+3rd+Noncoding. All positions containing gaps and missing data were eliminated. There were 421 positions in the final dataset. The different coloured circles represent the distribution of the phylogenetic groups. Red squares indicate the *S*. *coeruleoalba* haplotypes of the Gulf of Taranto (this study). Nodes labelled A-E indicate the clades discussed further in the text.

The evolutionary relationship between the 421 bp cyt b haplotype sequences was investigated by comparing *S*. *coeruleoalba* from the Gulf of Taranto with that from other geographical sites characterized by the available sequences corresponding to *S*. *clymene*, *S*. *frontalis*, *S*. *longirostris*, *S*. *attenuata*, *T*. *aduncus*, *T*. *truncatus*, and *D*. *delphis*. In particular, the following selection criterion was adopted. Only one gene sequence for each haplotype was included in the analysis of each species. All sequences were combined in the same alignment, and a phylogenetic tree was therefore constructed using the NJ method (Fig 3). The grouping in the tree was supported by high bootstrap probability values. The results of the analysis sustain, as expected, a species-specific clustering of the gene sequences from *S*. *frontalis*, *S*. *longirostris*, *S*. *attenuata*, *T*. *aduncus*, *T*. *truncatus*, and *D*. *delphis*, together with the polyphyletic distribution of the *S*. *clymene* sequences. Moreover the tree reveals a paraphyletic distribution of the *S*. *coeruleoalba* sequences Five clades are clearly recognizable (A to E) in these data. The clustering of the *S*. *coeruleoalba* sequences agrees with the pattern seen in the haplotype network (Fig 2). Particularly, there is a clear separation of the *S*. *coeruleoalba* haplotypes into two groups. One group is composed by most of the haplotypes from the Gulf of Taranto, indicating the possible distinctiveness of the Ionian population. In addition, there is a close relationship with the *T*. *truncatus* sequences. The other group produced results closer to the *D*. *delphis* haplotypes, with Hap 12 from *S*. *coeruleoalba* clustering with *D*. *delphis*. Moreover, Hap3 and Hap10 from *S*. *coeruleoalba* in the Gulf of Taranto are intermingled with sequences derived from other geographical areas. Looking at Hap 11 and Hap 13, it was possible to notice that *S*. *coeruleoalba*, *S*. *clymene* and *S*. *frontalis* are close sister taxa that are distant from *S*. *longirostris*, though still in the same subfamily. A maximun likelihood model applied to draw the phylogenetic tree gave the same arrangement of the sequences (S2 Fig)

## Discussion

In this paper we describe a skin swab method allowing tissue collection from swimming striped dolphins, *S*. *coeruleoalba*, in the Gulf of Taranto. We sequenced a 421 bp fragment of the mitochondrial cyt b gene and recovered 11 Haplotypes. We have analyzed the sequences from the study area and by comparing them with all sequences from other geographic sampling sites available in the database (19 Haplotypes), we have obtained a median-joining haplotype network.

The frequency of haplotypes observed in the Gulf of Taranto was comparable to that recorded in other geographical areas (i.e. NE Atlantic: haplotype numbers, 11 versus 9; sequences 25 versus 20); whereas the resulting nucleotide diversity was higher in the study area than in the other basins (i.e. NE Atlantic: nd 0.01990 versus 0.01176 (Tables 2 and 3).

As shown by the topology of the dendrogram, two groups were clearly detected within the samples from the Gulf of Taranto (Fig 2). A first compact group of 12 samples markedly differed from the second group of 11 samples. These latter samples were clearly connected with 7 samples collected in other Mediterranean areas (unknown location of Mediterranean Sea, Amaral, personal communication). The grouping of the eight haplotypes observed in the Gulf of Taranto (Hap 1, 2, 4, 5, 6, 7, 8, and 9) is separated by a consistent number of mutations from Hap 10, the most frequent haplotype in the Mediterranean Sea. Nevertheless, the geographic continuity between the Mediterranean Sea and NE Atlantic samples indicates the direct derivation of Hap 10 from Hap 29 (Coasts of Portugal; Amaral, personal communication), for which both the central position in the network and the frequency pattern (co-presence of samples from the Atlantic Ocean, Mediterranean Sea, and Indian Ocean), suggest the condition of ancestrality. To assess this relationship, a scenario with the following points can be depicted. An ancestor haplotype, as highly probably with Hap 29 from the North-Eastern Atlantic, could have entered the Mediterranean Sea and evolved into Hap 10. Subsequently, in the Gulf of Taranto (Northern Ionian Sea) several further mutations in the ancestral Hap 10, may have originated at least eight different haplotypes (Hap 1, 2, 4, 5, 6, 7, 8 and 9), but more likely a greater number of haplotypes.

Consequently, two groups of striped dolphins were genetically distinguishable in the study area. The former was characterized by the presence of Hap 10 as evidence of an ancestral condition. The latter group being characterized by its evolution (Hap 1, 2, 4, 5, 6, 7, 8 and 9). In an alternative scenario, it could be assumed that in the Gulf of Taranto, a population (green circles on the right side of the Fig 2) that derives from an ancestor different from the cosmopolitan one (Hap 10) exists as an independent lineage. Therefore our data suggest the presence of at least two different independent lineages of *S*. *coeruleoalba* in the Mediterranean Sea.

However, the availability of a small number of samples representative of the Mediterranean variability, and an even more limited number of samples representative of world-wide variability, requires a degree of caution in the data interpretation. Anyhow, the geomorphological and hydrographic characteristics of the Northern Ionian Sea population seem to support the hypothesis of a population of striped dolphin genetically diverging from its Mediterranean counterpart. In fact, the spatial distribution, abundance, and residency pattern of *S*. *coeruleoalba* in the study area was proven to be dependent on a critical habitat used by a population of striped dolphins for their day-to-day survival and health [22, 23, 24]. This habitat may have favoured the permanence of a population in the Gulf of Taranto without excluding the entry of cosmopolitan individuals from the rest of the Mediterranean Sea. Unfortunately, an intense human use of coastal and offshore areas in the Northern Ionian Sea highlights the urgent need for specific conservation measures in the Gulf of Taranto to reduce the probability of local extinction and address the risk of genetic erosion as indicated by the EU Marine Strategy Framework Directive (MSFD; Directive 2008/56/EC) and the UN Convention on Biological Diversity (CBD https://www.cbd.int) guidelines with respect to biodiversity.

Dolphins are the result of a rapid radiation occurring in the late Miocene that provided a diverse set of species. Molecular evolution of this group has been slow and demonstrates convergent evolution, hybridization, introgression of DNA from other species, and an incomplete lineage sorting that makes it difficult to assess their relationships. To this end, Perrin et al. [2] proposed temporary solutions to the existing problems of paraphyly among several genera, which would imply a reversion of all species within the subfamily Delphininae (i.e. *Stenella*, *Delphinus*, and *Tursiops*) back to *Delphinus*, the genus in which most were originally described. Phylogenetic analysis showed polyphyletic taxa, including *S*. *coeruleoalba*, *D*. *delphis*, *S*. *clymene*, *S*. *frontalis*, *T*. *truncatus*, and *T*. *aduncus*, with more than one recent common ancestor (Fig 3) according to that reported by LeDuc [6] and Amaral [7].

Unlike other findings, most of the striped dolphins sampled in the Gulf of Taranto (Northern Ionian Sea) were separated from the majority of *S*. *coeruleoalba* samples (node A), confirming the presence of at least two groups in the study area. One of these groups showed characteristics of a subpopulation. In fact, the striped dolphins sampled in the Northern Ionian Sea seem to be a monophyletic sister group with *T*. *truncatus* (node B). On the other hand, coherently with previous studies [2, 6], *S*. *coeruleoalba* in node C was more closely related to the genus *Delphinus*, with Hap 12 clustering together. In addition, two *S*. *coeruleoalba* sequences (Hap 11 and Hap 13) form a branch with their congeners *S*. *frontalis* and *S*. *clymene*, and with their paraphyletic taxon *T*. *aduncus* (node D). Additionally, *S*. *clymene* haplotype clustering agreed with the findings reported by Amaral et al. [45]. Particularly, the evolution of *S*. *clymene* may not follow a simple bifurcating tree, but likely derives from an admixture between two other closely related species, *S*. *coeruleoalba* and *S*. *longirostris*. Differently, *S*. *longirostris* and *S*. *attenuata* (Node E) form two polyphyletic groups that are separated from the rest of the genus *Stenella*.

In conclusion, the estimation of the our cyt b fragment data set with high bootstrap support probability values in NJ tree, highlights the loss of monophyly in *S*. *coeruleoalba*. This result is further supported by the median-joining haplotype network suggesting that *S*. *coeruleoalba* is oversplit as currently constituted. The group of haplotypes identified in the Gulf of Taranto diverges from the haplotypes of the Mediterranean Sea and from those of the more distant northeastern Atlantic, revealing the existence of an independent lineage. Moreover results suggest the need for specific measurements for the conservation of the striped dolphin in the Gulf of Taranto, reducing the probability of local *S*. *coeruleoalba* extinction and addressing the risk of genetic erosion according to the EU Marine Strategy Framework Directive and the UN Convention on Biological Diversity.

We hope that this report will be a starting point for future collaborations and for the development of databases focused on this area.

## Supporting information

S1 Table *Stenella coeruleoalba* cytochrome b haplotypes and the relative ENA ID. (PDF)

S2 Table Cytochrome b haplotype sequences used for the phylogenetic tree. (PDF)

S1 Fig Photograph of a specimen of striped dolphin. (JPG)

S2 Fig ML tree inferred from Delphinidae *cyt b* gene sequences. Evolutionary analyses were conducted in MEGA7 [42]. The evolutionary history was inferred by using the Maximum Likelihood method based on the Tamura-Nei model [41]. The tree is drawn to scale, with branch lengths measured in the number of substitutions per site. The analysis involved 94 nucleotide sequences. Codon positions included were 1st+2nd+3rd+Noncoding. All positions containing gaps and missing data were eliminated. There were a total of 421 positions in the final dataset.
(JPG)
