# Supplementary material for: Cytochrome b marker reveals an independent lineage of Stenella coeruleoalba in the Gulf of Taranto
Source: PLoS One. 2019 Mar 20;14(3):e0213826. doi: 10.1371/journal.pone.0213826 (PMC6426239; doi:10.1371/journal.pone.0213826)
Supplement: S1 Table — (PDF) [file pone.0213826.s001.pdf]

Table S1. *Stenella coeruleoalba* cytochrome b haplotypes and the relative ENA ID

Haplotypes	Accession numbers
Hap 1	LT971396
Hap 2	LT971397
Hap 3	LT971398
Hap 4	LT971399; LT971404
Hap 5	LT971400; LT971401; LT971402; LT971403
Hap 6	LT971405
Hap 7	LT971406
Hap 8	LT971407
Hap 9	LT971408
Hap 10	LT971409; LT971410; LT971411; LT971412; LT971413; LT971414; LT971415; LT971416; LT971417; LT971418; LT971419; KF691951; KF691963; KF691964; KF691966; KF692000; KF692005; KF692006
Hap 11	LT971420; KF692014
Hap 12	KF691978
Hap 13	KF691984; DQ466021
Hap 14	KF691996; DQ466022
Hap 15	KF692018; DQ466024
Hap 16	KF691992
Hap 17	KF692003; KF692001; KF691976
Hap 18	KF691959
Hap 19	KF691961
Hap 20	KF692015
Hap 21	KF692009; KF692008; KF691965; AF084082
Hap 22	KF691950
Hap 23	KF692017; DQ466018
Hap 24	KF692016; KF692011; DQ466017; EF090637
Hap 25	KF691962; DQ466016
Hap 26	KF692004
Hap 27	KF691999
Hap 28	KF691998; KF691997; KF691993; AF084081
Hap 29	KF692010; KF692007; KF692002; KF691960; DQ466023; DQ466020
Hap 30	EU557097; NC_012053

Acc. Numbers of the present work are from LT971396 to LT971420
