# Supplementary material for: Cytochrome b marker reveals an independent lineage of Stenella coeruleoalba in the Gulf of Taranto
Source: PLoS One. 2019 Mar 20;14(3):e0213826. doi: 10.1371/journal.pone.0213826 (PMC6426239; doi:10.1371/journal.pone.0213826)
Supplement: S2 Table — (PDF) [file pone.0213826.s002.pdf]

Table S2. Cytochrome b haplotype sequences used for the phylogenetic tree.

Haplotypes		Accession numbers
Stenella clymene	Ste_cly	KF692012; KF691985; KF691958; KF691994; KF692013; KF691986[1];
Stenella frontalis	Ste_fro	AF084089; AF084090
Stenella longirostris	Ste_lon	KF691954[2]; KF691980; KF691956; AF084102; KF691981[3];KF691977; KF691983; KF691975; X56293; X56292; KF691953; KF691957; KF691970; KF691952[4]; KF691968; KF691974; KF691967[5]
Stenella attenuata	Ste_att	AF084097; X56294; EU557096[6]; AF084096[7]; KX857269[8]
Tursiops aduncus	Tur_adu	AF084091[9]; AF084092[10];
Tursiops truncatus	Tur_tru	AF084093; AF084094; AF084095; DQ466025; DQ466026[11]; DQ466027[12]; DQ466028[13]; DQ466029; EU557093[14]
Delphinus delphis	Del_del	JX264697[15]; JX264703[16];JX264702; JX264599[17]; JX264690[18]; JX264696[19]; JX264616[20]; JX264700; JX264701[21]; JX264632; JX264601[22]; JX264611; JX264676[23]; JX264683[24]; KM225666; KM225667; KM225670; KC297765[25]; KC297766[26]; DQ378159; DQ378160; KM225664; KM225665

Accession Numbers sharing the same 421 bp sequence:

(1) from KF691986 to KF691991 as well as AF084083;
(2) KF691954, KF691958, KF691979 as well as AF084100;
(3) KF691981, KF691982 as well as AF084101;
(4) KF691952 and AF084103;
(5) KF691967, KF691969 and KF691971;
(6) EU557096 and NC012051;
(7) AF084096 and EF093030;
(8) KX857269, KX857273, KX857279, KX857280, KX857281, KX857286, KX857310, KX857314, KX857327, KX857333 and KX857341;
(9) AF084091, KF570335 and KF570337- KF570343;
(10) AF084092, AF084094, KF570389 and JN571479;
(11) DQ466026, KF570322, KF570326, KF570327, KF570332, KT601198 and MF669486;
(12) DQ466027, KF570320-21, KF570323-25, KF570328-30, KF570333-34, KT601188-89, KT601195, KT601200 and MF669485;
(13) DQ466028, KF570316-17, KF570319, KF570345-47, KF570349, KF570351-52, KT601190, KT601192-93
(14) EU557093, JN571478, JN571480, KF570379, KF570385-86 and KT601204;
(15) JX264697, JX264661, JX264652, JX264634, JX264628, JX264624, JX264621, JX264619, JX264614 and AF084085;
(16) JX264703, MF669498, MF669496, KM225671, KM225668, KM225661, KC297763, KC297759, KC297743, KC297739, KC297738, KC297736, KC297735, KC297722, JX264675, JX264673, JX264667, JX264639, JX264610, JX264596, DQ378161, DQ378155, DQ378153, DQ378150, DQ378148, DQ378139 and AF084084;
(17) JX264599, KC297725, KC297724, DQ378145, DQ378143 and DQ378141;
(18) JX264690, JX264663 and JX264631;
(19) JX264696, KM225663 and JX264633;
(20) JX264616, KC297742, JX264605, JX264603; JX264600, EF090636, DQ378164 and DQ378156;
(21) JX264701and JX264655;
(22) JX264601, KC297762 and DQ378157;
(23) JX264676, KC297761; KC297728, JX264607, JX264598, JX264595, JX264576, DQ378163, DQ378158, DQ378152, DQ378147, DQ378146 and DQ378140;
(24) JX264683 and JX264662;
(25) KC297765 and DQ378154;
(26) KC297766, KC297723, DQ378162 and DQ378142.
