# Supplementary figures and images for: Cytochrome b marker reveals an independent lineage of Stenella coeruleoalba in the Gulf of Taranto
Source: PLoS One. 2019 Mar 20;14(3):e0213826. doi: 10.1371/journal.pone.0213826 (PMC6426239; doi:10.1371/journal.pone.0213826)

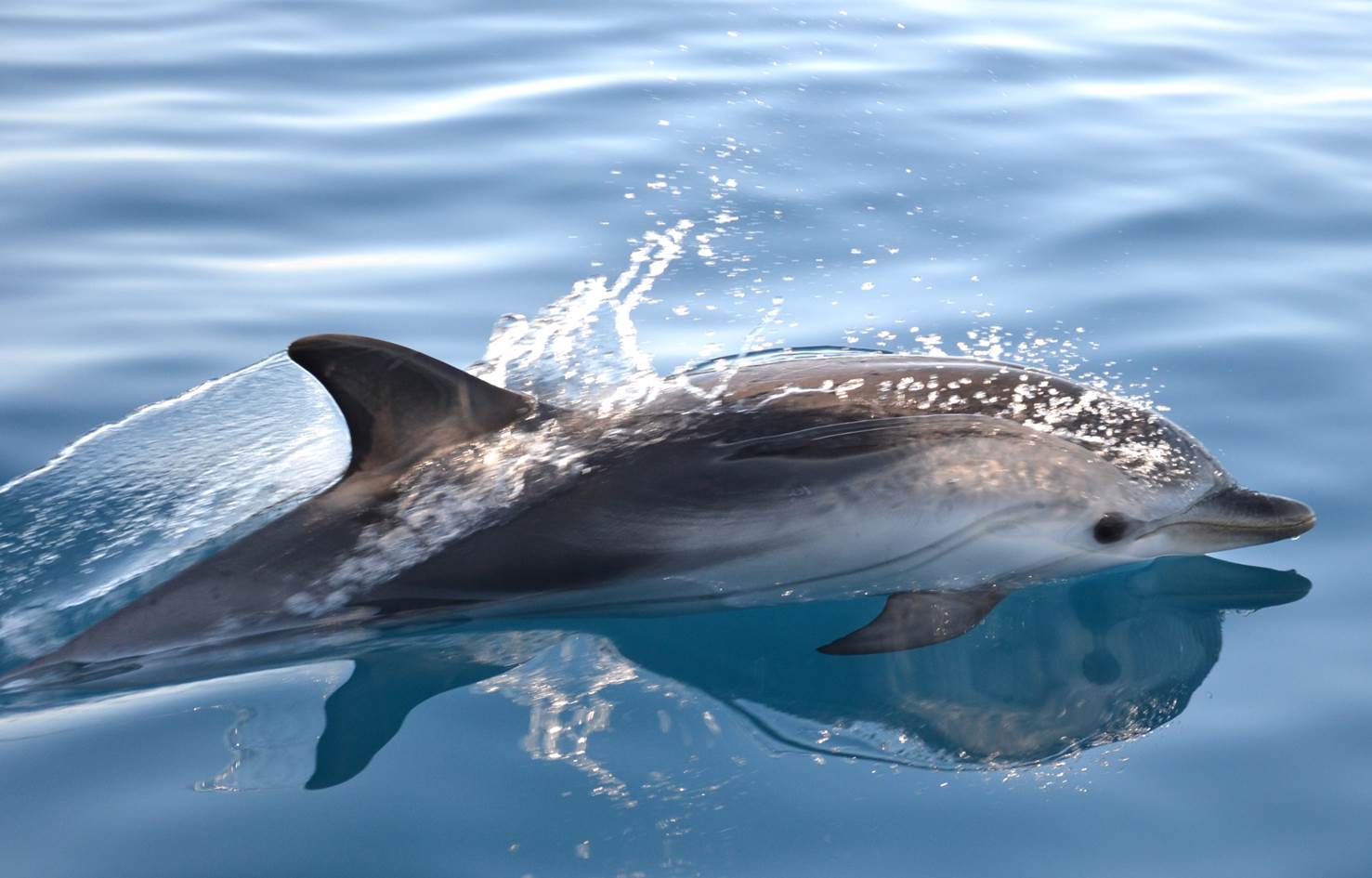

Supplement: S1 Fig — (JPG) [file pone.0213826.s003.jpg]

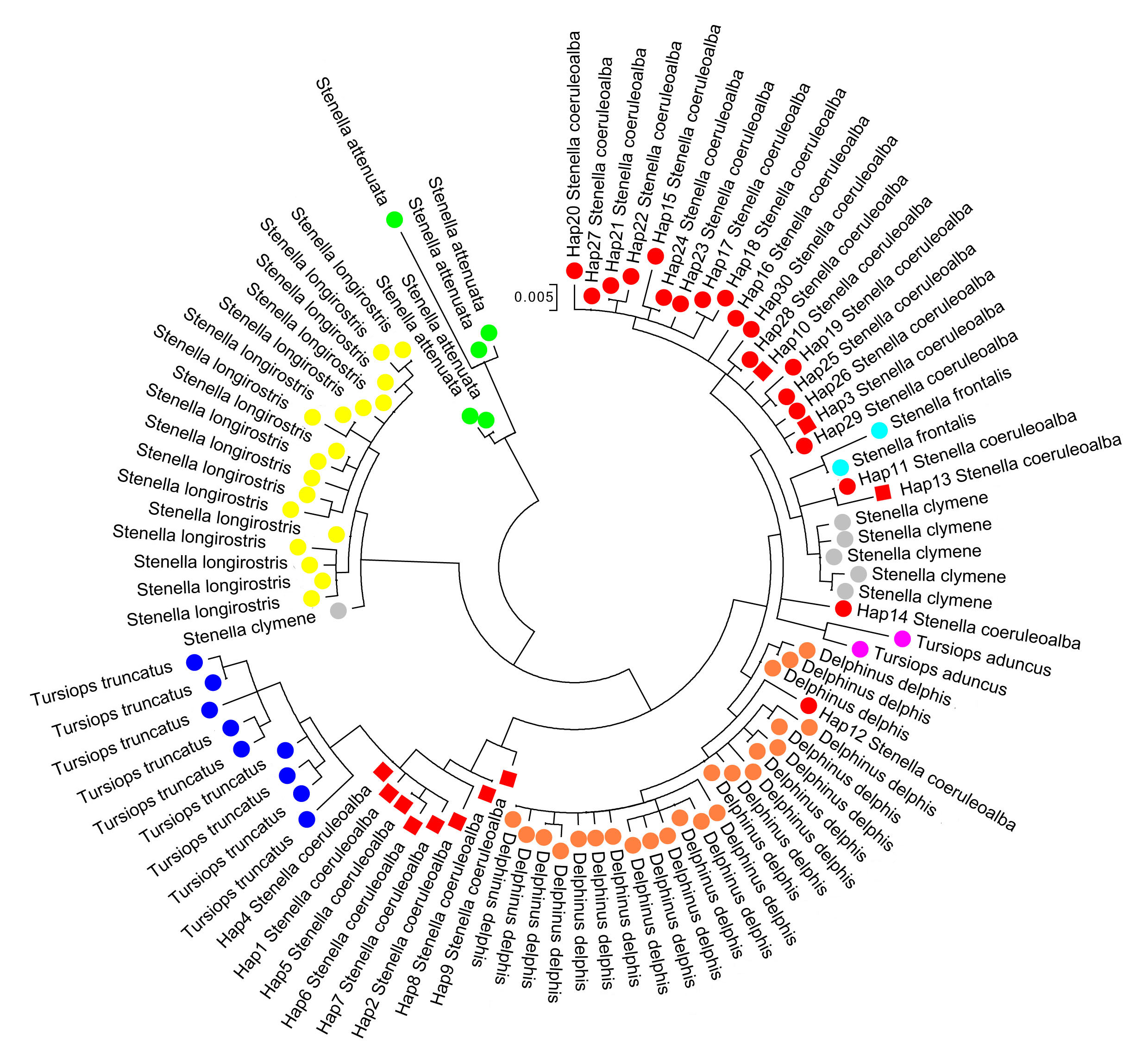

Supplement: S2 Fig — Evolutionary analyses were conducted in MEGA7 [42]. The evolutionary history was inferred by using the Maximum Likelihood method based on the Tamura-Nei model [41]. The tree is drawn to scale, with branch lengths measured in the number of substitutions per site. The analysis involved 94 nucleotide sequences. Codon positions included were 1st+2nd+3rd+Noncoding. All positions containing gaps and missing data were eliminated. There were a total of 421 positions in the final dataset. (JPG) [file pone.0213826.s004.jpg]
